# Effect of seasonal coronavirus immune imprinting on the immunogenicity of inactivated COVID-19 vaccination

**DOI:** 10.3389/fimmu.2023.1195533

**Published:** 2023-08-16

**Authors:** Di Yin, Zirong Han, Bing Lang, Yanjun Li, Guoqin Mai, Hongbiao Chen, Liqiang Feng, Yao-qing Chen, Huanle Luo, Yaming Xiong, Lin Jing, Xiangjun Du, Yuelong Shu, Caijun Sun

**Affiliations:** ^1^ School of Public Health (Shenzhen), Shenzhen Campus of Sun Yat-sen University, Shenzhen, China; ^2^ School of Public Health (Shenzhen), Sun Yat-sen University, Guangzhou, China; ^3^ Emergency Manage Department, Foshan, China; ^4^ Department of Epidemiology and Infectious Disease Control, Shenzhen, China; ^5^ State Key Laboratory of Respiratory Disease, Guangzhou Institutes of Biomedicine and Health (GIBH), Chinese Academy of Sciences, Guangzhou, China; ^6^ Institute of Clinical Medicine, First People's Hospital of Foshan, Foshan, China; ^7^ Key Laboratory of Tropical Disease Control (Sun Yat-sen University), Ministry of Education, Guangzhou, China; ^8^ National Health Commission of the People's Republic of China (NHC) Key Laboratory of System Biology of Pathogens, Institute of Pathogen Biology, Chinese Academy of Medical Sciences and Peking Union Medical College, Beijing, China

**Keywords:** SARS-CoV-2, seasonal coronavirus, immune imprinting, COVID-19 vaccination, pre-existing immunity

## Abstract

**Background:**

Pre-existing cross-reactive immunity among different coronaviruses, also termed immune imprinting, may have a comprehensive impact on subsequent SARS-CoV-2 infection and COVID-19 vaccination effectiveness. Here, we aim to explore the interplay between pre-existing seasonal coronaviruses (sCoVs) antibodies and the humoral immunity induced by COVID-19 vaccination.

**Methods:**

We first collected serum samples from healthy donors prior to COVID-19 pandemic and individuals who had received COVID-19 vaccination post-pandemic in China, and the levels of IgG antibodies against sCoVs and SARS-CoV-2 were detected by ELISA. Wilcoxon rank sum test and chi-square test were used to compare the difference in magnitude and seropositivity rate between two groups. Then, we recruited a longitudinal cohort to collect serum samples before and after COVID-19 vaccination. The levels of IgG antibodies against SARS-CoV-2 S, S1, S2 and N antigen were monitored. Association between pre-existing sCoVs antibody and COVID-19 vaccination-induced antibodies were analyzed by Spearman rank correlation.

**Results:**

96.0% samples (339/353) showed the presence of IgG antibodies against at least one subtype of sCoVs. 229E and OC43 exhibited the highest seroprevalence rates at 78.5% and 72.0%, respectively, followed by NL63 (60.9%) and HKU1 (52.4%). The levels of IgG antibodies against two β coronaviruses (OC43 and HKU1) were significantly higher in these donors who had inoculated with COVID-19 vaccines compared to pre-pandemic healthy donors. However, we found that COVID-19 vaccine-induced antibody levels were not significant different between two groups with high levelor low level of pre-existing sCoVs antibody among the longitudinal cohort.

**Conclusion:**

We found a high prevalence of antibodies against sCoVs in Chinese population. The immune imprinting by sCoVs could be reactivated by COVID-19 vaccination, but it did not appear to be a major factor affecting the immunogenicity of COVID-19 vaccine. These findings will provide insights into understanding the impact of immune imprinting on subsequent multiple shots of COVID-19 vaccines.

## 1 Introduction

Coronaviruses (CoVs) are a large family of enveloped, positive-stranded RNA viruses classified into four genera (alpha, beta, gamma, and delta) based on their phylogenetic and serological relationships (1). Human CoVs (hCoVs), belonging to alpha and beta genera, can cause a variety of symptoms ranging from the common cold to fatal pneumonia (2). Among them, four kinds of seasonal CoVs (sCoVs) including alpha CoVs (NL63 and 229E) and beta CoVs (OC43 and HKU1), are endemic worldwide and represent 10-30% of upper respiratory tract infections (1, 2). Previous studies demonstrated that most adults had been exposed to sCoVs, characteristic with seropositive responses to one or several sCoVs (3). However, immunity to these sCoVs is short-lived and wanes over time (4). The relative ratios of antibodies against any one of the four sCoVs are highly variable and dependent on the most recent exposure (5). So far, there is lack of data on the prevalence of antibodies to these four sCoVs in Chinese population.

Immune imprinting, refers to the preferential activation of memory B cells, that are generated during a prior infection with an antigenically related virus, rather than naive B cells specific for the novel virus (6, 7). This concept is well documented for influenza infections whereby humans are repeatedly exposed to antigenically distinct viruses containing homologous sequences. A recent study demonstrated that the immune imprinting by infections with the earlier wild-type SARS-CoV-2 or other variants resulted in less durable binding antibody against Omicron antigen (8). Studies also showed the presence of cross-reactive antibodies and T cells against SARS-CoV-2 by prior sCoVs infection (9, 10), implying that the phenomenon of immune imprinting might also exist between the different coronaviruses. Indeed, some studies demonstrated that the different levels of pre-existing sCoVs-specific antibodies might be correlated with the susceptibility and disease severity of SARS-CoV-2 infections (11, 12), and thus it is suggested that the prevalence of pre-existing sCoVs antibodies might be a potential factor to affect the discrepancy of COVID-19 pandemic and the effectiveness of COVID-19 vaccination among different regions (13, 14). However, the existing literature is still limited regarding the pre-existing sCoVs-specific antibodies and their potential influence on SARS-CoV-2 infection and COVID-19 vaccination effectiveness.

The inactivated COVID-19 vaccine, which has been extensively administered because of its safety and effectiveness (15, 16), may be influenced by various factors in its immunogenicity. Previous studies have indicated the existence of conserved epitopes between sCoVs and SARS-CoV-2 (17), which could be activated upon vaccination with the inactivated COVID-19 vaccine, leading to alterations in the magnitude and proportions of vaccine-induced immune responses. Therefore, exploring the mutual interactions between pre-existing immunity to sCoVs and the immune response induced by the inactivated COVID-19 vaccine remains to be further elucidated. As a result, this will contribute to a better understanding of the immunogenicity by the inactivated COVID-19 vaccine among different populations, thus guiding the promotion of inactivated COVID-19 vaccines and the design of next-generation COVID-19 vaccines.

In the present study, we assessed the seroprevalence and influencing factors of four sCoVs-specific antibodies, along with the cross-reactivity antibodies to the SARS-CoV-2, in the Chinese population before and after COVID-19 pandemic. Furthermore, we investigated how this immune imprinting will affect the immunogenicity of inactivated COVID-19 vaccines in a longitudinal cohort. These findings will provide insights into understanding the comprehensive interaction between immune imprinting of sCoVs and the immunogenicity of COVID-19 vaccination.

## 2 Methods

### 2.1 Sample collection

**Figure 1:**
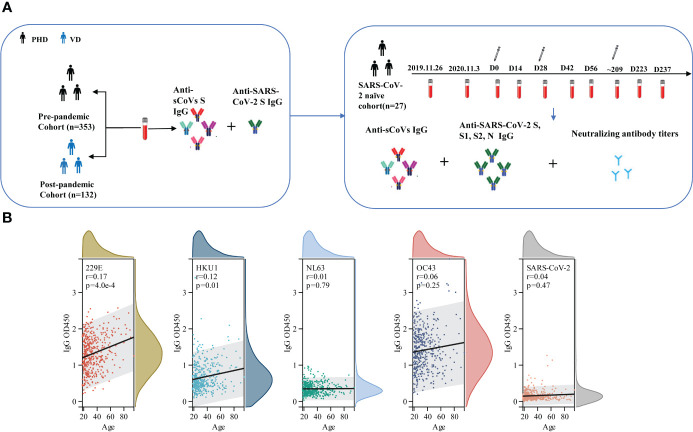
Schematic diagram of whole experimental design and the correlation between age and the level of pre-existing sCoVs antibodies. **(A)** schematic diagram to illuminate the whole experimental design. In brief, a cross-sectional study to collect serum samples (n=353) from healthy blood donors prior to the COVID-19 pandemic, a cross-sectional study to collect serum samples (n=132) from individuals who had received three doses of the inactivated COVID-19 vaccine post-pandemic, and a longitudinal cohort study (n=27) were included in our study. The levels of specific antibodies against sCoVs and SARS-CoV-2 spike in these samples were detected by ELISA and pseudotyped virus neutralization test. **(B)** Correlation between age and the level of pre-existing sCoVs antibodies in PHD. Wilcoxon rank sum test and chi-square test were used to compare the difference in magnitude and seropositivity rate between two groups. Spearman rank correlation was employed to test the correlation between variables. ns, no significance; PHD, pre-pandemic healthy donors; VD, vaccinated donors.

Schematic diagram of whole experimental design was shown in **Figure 1A**. A total of 353 serum samples from pre-pandemic healthy donors (PHD) across various age ranges were collected at blood donation center of Guangzhou and Shenzhen, China, in August 2019. 132 post-pandemic serum samples from donors who received three doses of inactivated COVID-19 vaccine (VD) were collected from April 21 to 22, 2022 at the physical examination center of First People’s Hospital of Foshan, China. SARS-CoV-2-negative infection was confirmed by reverse transcriptase polymerase chain reaction assays. Demographic information of PHD and VD, including age and gender, regarding these samples was recorded (**Table 1**).

**Table 1:** Demographic information of the included population^a^.

	Pre-pandemic healthy donors (PHD) (n= 353)	Vaccinated donors (VD) (n=132)	*P* value
*Mean age (yrs)*	35.2	38.8	0.302
Age groups (yrs)			
18-30	149 (42.2%)	48 (36.4%)	
31-45	99 (28.0%)	38 (28.8%)	
46-60	76 (21.5%)	34 (25.8%)	
>60	29 (8.2%)	12 (8.6%)	
Gender			
Female	169 (47.9%)	55 (41.7%)	0.211
Male	184 (52.1%)	77 (58.3%)	

^a^Percentages for some items may not sum up to 100 percent due to rounding.

The longitudinal cohort was recruited from healthy donors at Shenzhen campus of Sun Yat-sen University in China. We collected 242 serum samples from 27 participants on 26 November 2019, 13 November 2020, and 10 April 2022 before immunization with the inactivated COVID-19 vaccine and on days 14 and 28 after each time of vaccination. All of the participants completed three doses of CoronaVac (inactivated SARS-CoV-2 vaccine). As of the day of sampling, none of them had reported being positive for SARS-CoV-2 nucleic acid test. Demographic information, including age and gender, was also recorded (**Supplementary Table 1**).

All blood samples were collected in 2 ml disposable serum tubes (Jet, SST001020). After centrifugation at 6000g for 10 minutes, the serum was separated and incubated at 56°C for 30 minutes to inactivate the potential pathogens, and then used for the subsequent Enzyme-Linked Immunosorbent Assay (ELISA) assays.

### 2.2 Detection of IgG antibodies by ELISA

Antigen-specific IgG antibodies were analyzed using a standard ELISA procedure as previously described (18). Briefly, to detect corresponding antibodies, 96-well EIA/RIA plates (Corning, #3590) were coated with recombinant SARS-CoV-2 full-length S (Sino Biological, #40591-V08B1-1), SARS-CoV-2 S1(Sino Biological, #40591-V08H), S2 (Sino Biological, #40590-V08B), SARS-CoV-2 full-length N (Sino Biological, #40588-V08B), HCoV-HKU1 full-length S (Sino Biological, #40606-V08B), HCoV-NL63 full-length S (Sino Biological, #40604-V08B), HCoV-OC43 full-length S (Sino Biological, #40607-V08B), and HCoV-229E full-length S proteins (Sino Biological, #40605-V08B) at 100ng/ul in ELISA coating buffer (Solarbio, #C1055) respectively, and incubated at 4°C overnight. After washing three times with phosphate-buffered saline (PBS, PH=7.4), the plates were blocked with PBS/0.1%Tween (PBST) containing 5% nonfat-dried milk (Blocking solution) for 1.5-2 hours at 37°C. Then, serum samples were diluted in blocking solution (1:200) and added to the corresponding wells. Afterward, plates were incubated for 2 hours at 37°C. The reference serum sample was also incubated at a serial 2-fold dilution from 1: 50 to 1: 6400 at the same time. After washing three times with PBST, Horseradish Peroxidase (HRP)-conjugated anti-human IgG antibody (Abbkine, #A21050) was added after diluted in blocking solution (1:10000). Then, plates were incubated for 1.5 hours at 37°C. After washing six times with PBST, the substrate (Millipore, #ES001-500ML) was added (50 μl/well) in the dark for 25 minutes. Thereafter, the reaction was stopped with ELISA termination solution (Solarbio, #C1058). The optical density (OD) was measured at 450nm by Multimode reader (Biotek).

### 2.3 Definition of reference samples and calculation of cut-off values

A major barrier to estimate the seroprevalence of four sCoVs-specific antibodies is the absent of true negative reference population. To overcome this challenge, we conducted a preliminary test on additional pre-pandemic samples using ELISA. Besides, we developed and optimized an approach by cell-based flow cytometry to assess the binding ability for serum antibodies and coronaviruses S protein expressed onto the cell surface as described previously (19). In brief, we constructed the recombinant plasmids containing the full-length spike genes of SARS-CoV-2 and four kinds of sCoVs, respectively. Subsequently, 2×10^4^ 293T cells per well were transfected with these recombinant plasmids in a 96-well plate and incubated at 37°C for 36-48 hours, allowing for the expression of the respective S protein on the surface of the 293T cells. Following this, sera samples were added in 1: 200 dilution and incubated for 1 hour at 37°C. The plates were washed twice with FACS buffer and then stained with 50 μl/well of 1: 100 dilution of R-phycoerythrin (PE)-conjugated mouse anti-human IgG Fc antibody (SouthernBiotech, #9040-09) on ice in dark for 45min. After two additional washes, stained cells were analyzed using flow cytometry (Beckman, Cytoflex), and the binding data were generated by calculating the percent (%) of PE-positive cells for antigen binding using CytoExpert software.

Only those serum samples that show undetectable OD450 values in the ELISA pre-test and have no or weak positive PE fluorescence signal in the following cell-based flow cytometry will be considered as negative reference (**Supplementary Figure 1**). Based on the aforementioned pre-experiment, three negative control samples for each antigen of the four sCoVs and SARS-CoV-2 were defined. The cutoff value was determined by calculating the mean of three negative references plus 3 standard deviations (SD). In addition, the serum samples containing high antibody titers against four kinds of sCoVs in the pre-experiment were used as positive reference. A convalescent serum sample from a subject with confirmed SARS-CoV-2 infection was used as positive reference standards for antibodies against SARS-CoV-2.

### 2.4 Pseudotyped virus neutralization test

To generate the SARS-CoV-2 spike pseudotyped virus, we co-transfected 60 μg of plasmid pNL4-3.luc.RE and 20 μg of various SARS-CoV-2 spike variants into HEK293T cells in 15 cm cell culture dishes using PEI. The supernatant was harvested 72 h post transfection, centrifuged at 1000 rpm and stored at –80 °C until use. To assess the neutralizing activity, we performed the PVNT following our previously described method (20). Plasma samples were serially diluted and incubated with an equal volume of 200 (TCID50) of SARS-Co pseudovirus into 96 well-plate at 37°C for 1 hour. Subsequently, 6-8×10^4^ HEK293T-hACE2 cells were added to the plates. After 48 h incubation at 37°C, the supernatant was removed, and 150 μL of cell lysate was added to the cells. Following a 15 minutes incubation at RT, 50 μl of Firefly luciferase substrate was transferred to 96-well white solid plates for measurements of luminescence using the promegaGlomax chemiluminescence analyzer (Promega). The neutralizing antibody titers (NT50) was calculated using GraphPad Prism 8.0 software employing the log (inhibitor) vs. normalized response-Variable slope (four parameters) model.

### 2.5 Statistical analysis

Mann–Whitney U test and Kruskal-Wallis test were employed to compare the differences in the non-parametric observations between different groups. Pearson’s chi-square test was performed to examine the differences in the antibody seroprevalence. The Spearman rank correlation coefficient was used to assess the degree of correlation between the level of antibodies. Statistical significance was defined as *p* < 0.05 and all hypothesis testing were two-tailed. Statistical analyses and figure rendering were conducted using SPSS version 25.0 and GraphPad Prism version 8.01. Correlation matrix analyses were performed using the Sangerbox tools (http://sangerbox.com).

### 2.6 Ethics declarations

This study was approved by the Ethics Committee of the School of Public Health (Shenzhen), Sun Yat-sen University (Approval number: SYSU-PHS-IACUC-2022 -019), and the human blood samples were taken with written informed consent from the volunteers.

## 3 Results

### 3.1 High prevalence of sCoVs-specific antibodies among Chinese population

We conducted a serological survey in Guangzhou and Shenzhen of China, to evaluate the prevalence of sCoVs among age groups in Chinese population before COVID-19 pandemic. The majority of these samples (339/353, 96.0%) showed the presence of IgG antibodies against at least one subtype of sCoVs. Among the different sCoVs subtypes, 229E and OC43 exhibited the highest seroprevalence rates at 78.5% and 72.0%, respectively, followed by NL63 (60.9%) and HKU1 (52.4%) (**Table 1**). These findings indicated a significant prevalence of sCoVs among Chinese population.

Then, we assessed the association between age, gender and the magnitude of sCoVs antibodies. Our analysis revealed that increasing age was correlated with higher antibody levels against HKU1 and 229E S (**Figure 1B**), supporting the notion that elderly individuals may have a higher seroprevalence of sCoVs. Furthermore, we found no significant correlation between gender and antibody levels (**Supplementary Table 2**).

### 3.2 Correlation between prior sCoVs infections and the cross-reactive antibodies against SARS-CoV-2

**Figure 2:**
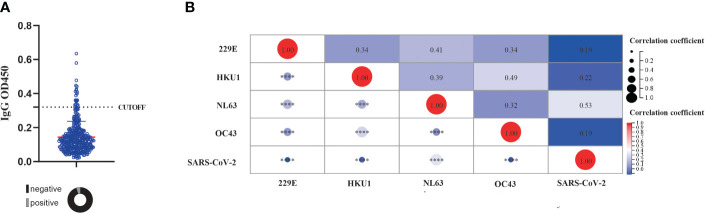
The presence of cross reactive antibody against SARS-CoV-2and its correlation with pre-existing sCoVs antibodies. **(A)** The detection of cross reactive antibody against SARS-CoV-2 in unexposed individuals. The dashed line represented the CUT-OFF value, which is calculated as the mean OD450 value of three negative controls plus 3 standard deviations. Wilcoxon rank sum test and chi-square test were used to compare the difference in magnitude and seropositivity rate between two groups. **(B)** Correlation of antibody levels against four kinds of sCoVs and SARS-CoV-2 spike in PHD. Spearman rank correlation was employed to test the correlation between variables. ****p < 0.0001.

We then investigated the presence of cross-immunity between sCoVs and SARS-CoV-2. We evaluated the seroprevalence of cross-reactive IgG antibodies binding to the SARS-CoV-2 spike protein in individuals who had not been exposed to the virus. Since we obtained serum samples prior to the COVID-19 outbreak, it ensured that these individuals had not been exposed to SARS-CoV-2. Consistent with previous studies (21), we identified the presence of cross-reactive antibodies against SARS-CoV-2 in the pre-pandemic healthy donors (PHD), with a seroprevalence rate of 5.4% (**Figure 2A** and **Table 1**). Given the substantial prevalence of sCoVs, the presence of these cross-reactive antibodies in the samples prior to SARS-CoV-2 exposure, might be a result of previous sCoVs infections.

To further investigate this phenomenon, we explored the relationship between pre-existing IgG antibodies against the spike antigen of SARS-CoV-2 and sCoVs. Our analysis revealed a significant association between the levels of IgG antibodies against the SARS-CoV-2 spike protein and the levels of IgG antibodies against four sCoVs (**Figure 2B**). This suggested a potential link between prior sCoVs infections and the generation of cross-reactive antibodies against SARS-CoV-2.

### 3.3 Reactivation of sCoVs immune imprinting by COVID-19 vaccination

**Figure 3:**
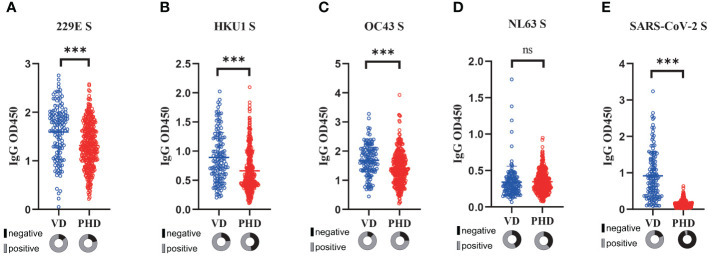
Reactivation of sCoVs immune imprinting by COVID-19 vaccination. Serum samples from healthy individuals (n=132) who had received three doses of inactivated COVID-19 vaccine were collected, and then the levels of antibodies against four kinds of sCoVs **(A-D)** and SARS-CoV-2 spike **(E)** were detected in VD (blue hollow circles) and PHD (red hollow circles) respectively. In the pie chart under the scatter plot, the seropositivity rate was shown in gray, while the seronegative rate was shown in black. Wilcoxon rank sum test and chi-square test were used to compare the difference in magnitude and seropositivity rate between two groups. *** *p* < 0.001. ns: no significance.

Next, we explored how the COVID-19 vaccination might affect the pre-existing sCoVs-specific immunity. To achieve this, we collected serum samples from 132 healthy individuals who had received three doses of inactivated COVID-19 vaccine, and then compared the levels of sCoVs-specific antibodies between these vaccinated donors (VD) and PHD. Both cohorts were gender-balanced and had similar mean ages (**Table 1**). We found that the magnitude of antibodies against HKU1, OC43, and 229E S was significantly higher in the donors who received the inactivated COVID-19 vaccine compared to the pre-pandemic healthy donors (**Figure 3**). This observation suggested that the reactivation of pre-existing immunity against β sCoVs may be triggered by the COVID-19 vaccination.

### 3.4 Pre-existing sCoVs antibodies scarcely affected the humoral immunity induced by COVID-19 vaccination

**Figure 4:**
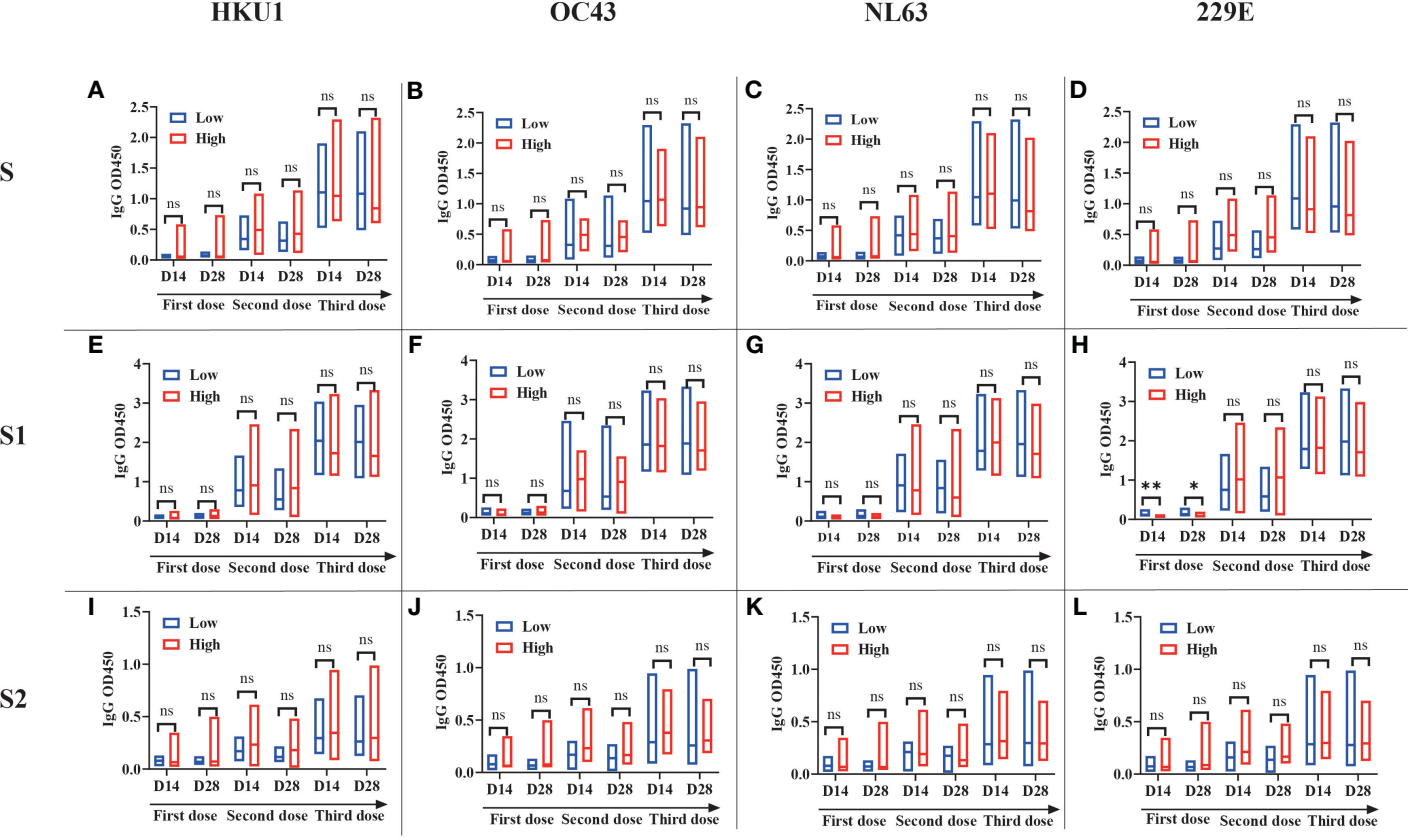
Pre-existing sCoVs antibodies scarcely affected the humoral immunity induced by COVID-19 vaccination A longitudinal study (n=27) was performed over a period of approximately two years. Based on the median magnitude of IgG antibodies against four kinds of sCoVs before the first dose of vaccination (Day0), we divided these participants into two groups, low level (represented by blue borders) and high level (represented by red borders) of pre-existing sCoVs antibodies. The IgG antibody profiles of SARS-CoV-2 S **(A-D)**, S1 **(E-H)** and S2 **(I-L)** at different time points were monitored before and after COVID-19 vaccination between the two groups. The data is presented in the form of a box plot, with the upper and lower bounds corresponding to 95% and 5% of the population, respectively. The solid line in the middle represents the position of the median. Wilcoxon rank sum test was used to compare the difference in magnitude between two groups. ns, no significance.

**Figure 5:**
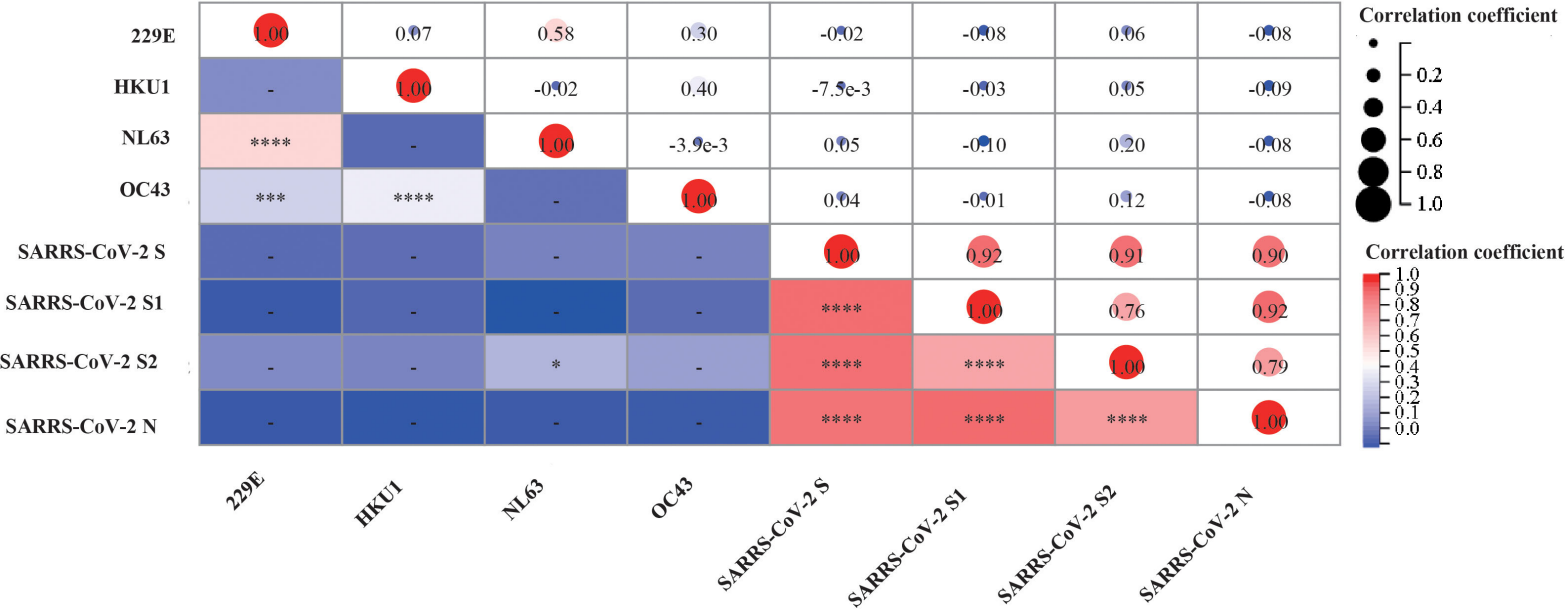
Correlation between the levels of antibody against sCoVs and SARS-CoV-2 after COVID-19 vaccination. We collected serum samples from participants and measured the levels of antibodies against sCoVs and SARS-CoV-2 on the 14th and 28th days after each time of COVID-19 vaccination. Spearman rank correlation was employed to test the potential correlation. In the lower left part of the correlation matrix, the color in each grid towards darker red represented a stronger correlation between the respective two variables. In the upper right part of the correlation matrix, the numbers in each grid represented the p-values of Spearman rank correlation for the respective two variables. *p < 0.05, ***p < 0.001, ****p < 0.0001.

To investigate the interaction between pre-existing sCoVs immunity and the inactivated COVID-19 vaccine-induced humoral immunity, we conducted a longitudinal study involving 27 participants over a period of approximately 2 years. We analyzed the IgG antibody profiles of sCoVs and SARS-CoV-2 before and after COVID-19 vaccination. Prior to vaccination, the seroprevalence of pre-existing antibodies against SARS-CoV-2 S, S1, S2, and N proteins ranged from 3.4% to 11.0%. Based on the median magnitude of IgG antibodies against four kinds of sCoVs before the first dose of vaccination (Day0), we divided these participants into two groups, high level and low level of pre-existing sCoVs antibodies, and then the levels of SARS-CoV-2 specific antibodies after vaccination between the two groups were compared. We found that at most time points, the levels of pre-existing sCoVs antibodies did not affect the magnitudes of inactivated COVID-19 vaccine-induced antibodies (**Figure 4**). The correlation analysis was also consistent with this observation, except for a weak correlation found between NL63 S and SARS-CoV-2 S2 IgG antibody levels (**Figure 5**).

**Figure 6:**
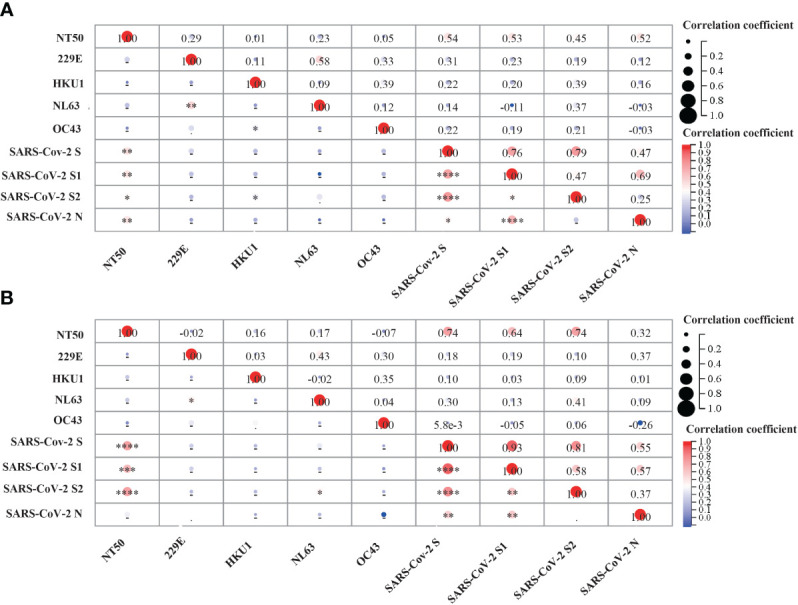
Correlation analysis between pre-existing sCoVs IgG antibodies, SARS-CoV-2 IgG antibodies and SARS-CoV-2 neutralizing antibody titer (NT50).SARS-CoV-2 neutralizing antibody titer (NT50) was detected on the 28th day after COVID-19 vaccination. The correlation matrix represented the potential correlation between the NT50 values and IgG antibody levels against sCoVs and SARS-CoV-2 in the serum after the 2nd dose **(A)** and the 3rd dose **(B)** of COVID-19 vaccination. Spearman rank correlation was employed to test the correlation between variables. In the lower left part of the correlation matrix, the color in each grid towards darker red represented a stronger correlation between the respective two variables. In the upper right part of the correlation matrix, the numbers in each grid represented the p-values of Spearman rank correlation for the respective two variables. *p < 0.05, **p < 0.01, ***p < 0.001, ****p < 0.0001.

Previous studies have also detected neutralizing activity against SARS-CoV-2 in pre-pandemic cohorts (22), suggesting that prior sCoVs infection may have developed memory immune responses against neutralizing epitopes of SARS-CoV-2 in some unexposed individuals. Thus, we investigated whether these sCoVs antibodies influenced the COVID-19 vaccine-induced neutralizing activity. We measured antibody levels in the serum of participants on day 28 after the second and third doses of vaccination. While a significant correlation was observed between the neutralization titer and the levels of antibodies against SARS-CoV-2, no correlation was found between the levels of sCoVs antibodies and the neutralization titer (**Figures 6A, B**).

In summary, our results indicated that pre-existing sCoVs antibodies did not appear to be a major factor affecting the inactivated COVID-19 vaccine-induced antibody responses. **Table 2**

**Table 2:** Seroprevalence of sCoVs and SARS-CoV-2 S IgG antibodies in different cohorts.

Seroprevalence (%)	Pre-pandemic healthy donors (PHD) (n= 353)	Vaccinated donors (VD) (n=132)	*P* value
229E S	78.5	85.6	0.078
HKU1 S	52.4	76.5	<0.001
OC43 S	72.0	87.9	<0.001
NL63 S	60.9	56.8	0.414
SARS-COV-2 S	5.4	89.0	<0.001

## 4 Discussion

In the present study, the seroprevalence of sCoVs in healthy donors were investigated in southern China. Among the subtype of sCoVs, 229E and OC43 were the most widely distributed, with seroprevalence of 78.5% and 75.1%, respectively. These findings are comparable to another study conducted in Canada that showed the seroprevalence of 82.9% for OC43 and 82.1% for 229E (22).Considering the high prevalence of anti-sCoVs immunity in the general population,immune imprinting should be an important issue to further optimize the strategy for the mass vaccination against SARS-CoV-2 infections (23–25). However, the interaction between pre-existing anti-sCoVs immunity and SARS-CoV-2 specific immune responses remains elusive.

Increasing evidence have suggested that age may play a role in the seroprevalence distribution of sCoVs-specific antibodies (26). Our study also supported the idea that elderly individuals have a higher seroprevalence of sCoVs (27). These age-dependent differences could be explained by the repeated sCoVs exposure with age. Furthermore, the immune system in the elderly people can be compromised due to aging or immunosenescence, which could lead to more frequent reinfections with sCoVs. With each exposure, memory B cells undergo affinity maturation and clonal selection, resulting in the generation of higher-affinity sCoVs-specific antibodies. Consequently, as individuals age, the repeated exposure to sCoVs can cause a more specific and less adaptable repertoire of sCoVs-specific memory B cells.

Previous studies have demonstrated the presence of immune imprinting in influenza infections (28) and coronaviruses infections (23). Overall, the four sCoVs shared 23% to 30% of sequence homology with the spike of SARS-CoV-2 (17), and the conserved epitopes located in the spike S2 domain and nucleocapsid are thought to be responsible for cross-reactive immunity between sCoVs and SARS-CoV-2. In line with these findings, we found cross-reactive IgG antibody against SARS-CoV-2 spike in 5.6% unexposed individuals. These data were also consistent with previous studies of pre-pandemic cohorts, which reported that 1.2%-5.3% of sera samples displayed IgG reactivity against the SARS-CoV-2 S antigen (21, 29). Our findings further supported the existence of immune imprinting resulting from prior sCoVs infections. These immune imprinting may have implications at the population level for susceptibility and disease severity of SARS-CoV-2. However, recent efforts on this aspect have yielded controversial results (11, 30). One possible reason for the differences among these studies may be lack of continuity and comparability of sample sources. Besides, different antigens of the coronaviruses involved in these studies may also cause this discrepancy. So far, the roles of cross-reactive antibodies are complicated. Studies have demonstrated that the cross-reactive non-neutralizing antibodies might restrict SARS-CoV-2 infection through antibody-dependent cell-mediated cytotoxicity (ADCC) and complement-fixing by binding to viral protein, which is expressed on the surface of SARS-CoV-2 infected cells (31). On the other hand, abundant non-neutralizing antibodies might also promote viral infection through Fc receptor binding, and thus leading to the antibody-dependent enhancement (ADE) (32). Given the extensive presence of pre-existing cross-reactive immunity to SARS-CoV-2 among the different populations, the immune imprinting caused by sCoVs may have significant implications for the subsequent population immunity against the SARS-CoV-2. Clarifying these effects will be beneficial in gaining a better understanding of SARS-CoV-2 evolution, population-level susceptibility, and the variations in the protective efficacy of COVID-19 vaccines among different population.

An unresolved question is how immune imprinting will affect the immunogenicity of COVID-19 vaccines. Although several studies reported that immune imprinting may impair the immunogenicity of influenza vaccines (33, 34), limited research has investigated how this immune imprinting will affect the immunogenicity of COVID-19 vaccines. Notably, a recent study found that when compared with infection-naïve vaccinated individuals and unvaccinated COVID-19 convalescent individuals, those vaccinated convalescent individuals had the highest concentrations of Spike–specific antibodies (35), indicating that prior SRAS-CoV-2 infection might enhance the immunogenicity of COVID-19 vaccine.sCoVs and SRAS-CoV-2 both belong to the coronavirus and share certain genetic homology. Given the wide spread of sCoVs among the populations, we investigated whether prior sCoVs infection would impact the immunogenicity of the inactivated COVID-19 vaccine. Consistent with one previous study (36), our findings indicated that sCoVs antibody levels scarcely affected the neutralizing antibody titer induced by the inactivated vaccine, suggesting that immune imprinting induced by prior sCoVs infection did not appear to be a major factor affecting the immunogenicity of inactivated COVID-19 vaccine. This may be attributed to the distribution of cross-reactive epitopes between sCoVs and SARS-CoV-2, as pre-existing cross-reactive antibodies triggered by sCoVs infections primarily target non-neutralizing sites of SARS-CoV-2 (19, 30). Moreover, although a broadly neutralizing antibody against SARS-CoV-2 was screened from the serum of SARS-CoV patients (37), little SARS-CoV-2 neutralizing antibody was found from pre-pandemic sera (38, 39). These studies suggested that the memory immune response against neutralizing epitopes induced by sCoVs infection is rarely reactivated by subsequent immunization of COVID-19 vaccine.

With the repeated infections of sCoVs and SARS-CoV-2 variants or the different doses of COVID-19 vaccination, the increasingly pre-existing cross-reactive immunity against the SARS-CoV-2 among different populations and the concept of “hybrid immunity” will be garnered considerable attention (40, 41). Of note, these immune responses targeting the conserved epitopes between sCoVs and SARS-CoV-2 variants might gradually occupy a growing proportion in the reservoir of memory immune cells. Consequently, for the design of the next generation of COVID-19 vaccines, it will be important to determine how to effectively utilize or avoid the impact of these pre-existing immune responses. For example, T-cell responses targeting the conserved antigens have been found to be immunogenic and effective against the severe COVID-19 symptoms (42, 43), indicating that these conserved T epitopes may be considered as antigen candidates to induce a broad-spectrum protective immunity among different coronavirus. In addition, the occurrence of immune imprinting in mice can be mitigated by administering specific dendritic cell-activating adjuvants during either the initial or subsequent antigen exposures (44) and thus it is necessary to develop novel vaccine adjuvants to overcome the impact of immune imprinting and thereby enhance the immunogenicity and effectiveness of the COVID-19 vaccines.

There are some limitations in our study. Firstly, our serological investigation did not have a sampling process, which may affect the representativeness of the different population. However, it is worth noting that the demographic characteristics of serum donors before and after the pandemic were adjusted to a comparable level, which increases the reliability of our results. Second, we only detected the antibody level in this study, but the effect of pre-existing cross-reactive T cells on COVID-19 vaccine was not explored. Given the extensive distribution of pre-existing cross-reactive T cells in populations (9, 45), it’s an important issue to further investigate the interaction between pre-existing sCoVs immune imprinting and the COVID-19 vaccine-induced T-cell responses.

## 5 Conclusions

We found a high prevalence of antibodies against four sCoVs in healthy donors in Chinese population, and the majority of donors contained pre-existing antibodies specific against at least one sbutype of sCoVs. We also found that the seroprevalence and antibody titers against sCoVs were higher in inactivated vaccine recipients than that in healthy donors, implying that pre-existing immunity to β sCoVs might be effectively reactivated by theCOVID-19 vaccination. In addition, we found that prior sCoVs infection scarcely affect the humoral immune response induced by inactivated COVID-19 vaccine. Thus, our results support the widespread promotion of inactivated COVID-19 vaccines across the entire population, particularly for the elderly and children, who usually possess a higher level of pre-existing sCoV antibodies. In addition, given the complexity and diversity of population’s immunological background, each individual’s immune imprinting may vary, which could potentially hinder the application of vaccines. For the design of the next generation of COVID-19 vaccines, we should pay more attention on the impact of immune imprinting due to the repeated infections of different coronaviruses or the different shots of COVID-19 vaccines.

## Data availability statement

The raw data supporting the conclusions of this article will be made available by the authors, without undue reservation.

## Ethics statement

The studies involving humans were approved by Ethics Committee of the School of Public Health (Shenzhen), Sun Yat-sen University. The studies were conducted in accordance with the local legislation and institutional requirements. The participants provided their written informed consent to participate in this study.

## Author contributions

Project design and supervised by CS, DY; experiment performing by DY, ZH,, BL; data analysis by DY, ZH, BL; materials and reagents contributed by GM, YS, YX, XD, LF, HC, HL, Y-QC, YL; writing by DY, ZH; review and editing by CS. All authors have read and agreed to the published version of the manuscript.

## Supplementary material

Supplementary Table 1 Demographic and sampling information for the longitudinal cohort ^a^ One participant missed a sampling due to uncontrollable factors

Supplementary Table 2
*P* values of Spearman rank test for sCoVs and SARS-CoV-2 S IgG antibodies grouped according to the gender across different populations
