# Supplementary material for: Effect of seasonal coronavirus immune imprinting on the immunogenicity of inactivated COVID-19 vaccination
Source: Front Immunol. 2023 Aug 16;14:1195533. doi: 10.3389/fimmu.2023.1195533 (PMC10467281; doi:10.3389/fimmu.2023.1195533)
Supplement: Supplementary Table 1 — Demographic and sampling information for the longitudinal cohort a One participant missed a sampling due to uncontrollable factors [file DataSheet_1.pdf]

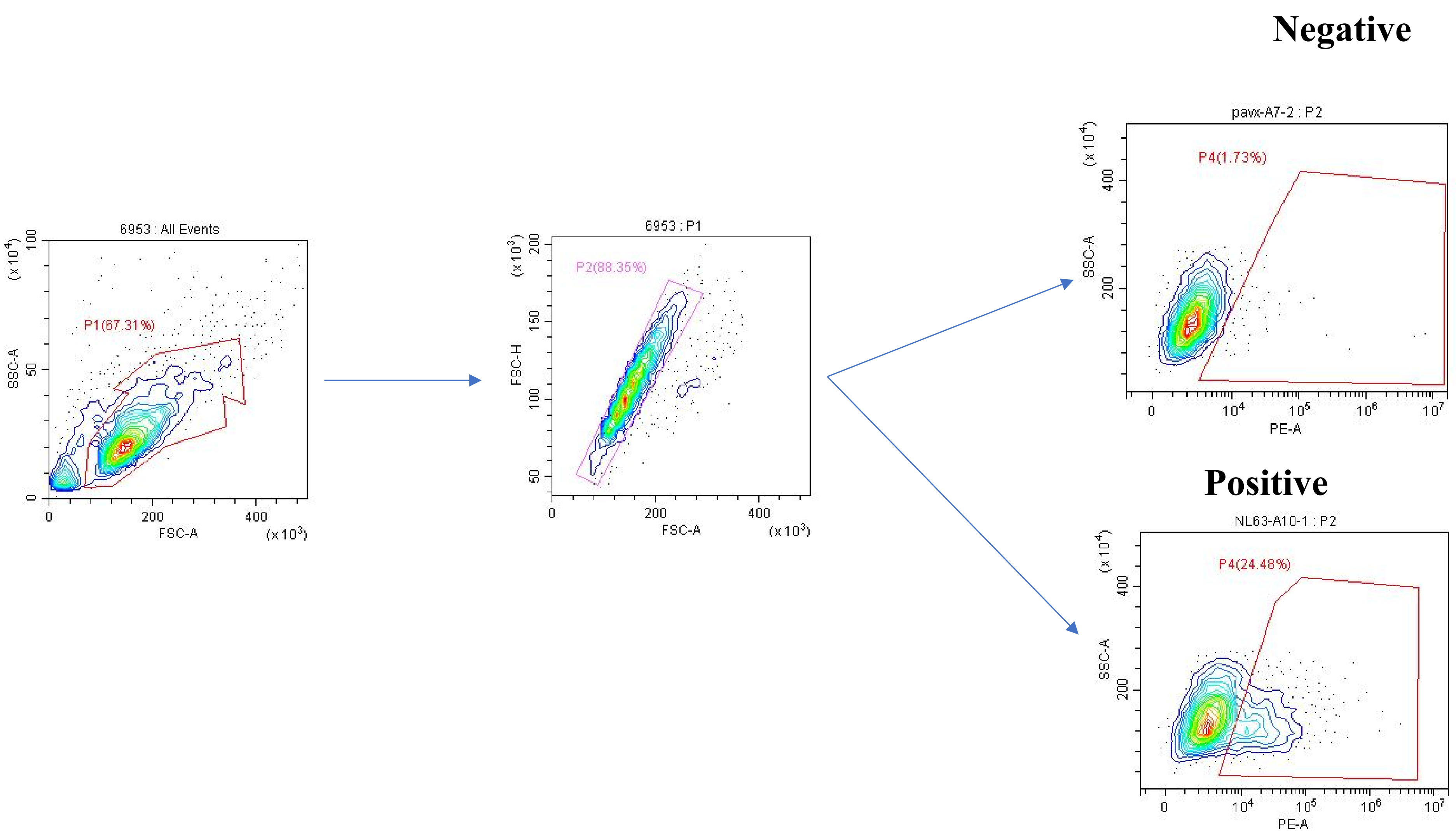

Supplementary Figure 1. Example gating strategy for flow cytometry . Data acquired on the Beckman flowcyto and analyzed on Cytoexpert software.

Supplementary Table 1 Demographic and sampling information for the longitudinal cohort

Sampling point in time	n[a]	Mean age (yrs)	Gender (Male/ Female)
2019.11.26	27	18.3	11/16
2020.11.3	27	18.3	11/16
2021.4.10	27	18.3	11/16
2021.5.6	26	18.3	10/16
2021.5.20	27	18.3	11/16
2021.6.3	27	18.3	11/16
2021.6.17	27	18.3	11/16
2021.12.15	27	18.3	11/16
2021.12.29	27	18.3	11/16

[a] One participant missed a sampling due to uncontrollable factors

Supplementary Table 2 *P* values of Spearman rank test for sCoVs and SARS-CoV-2 S IgG antibodies grouped according to the gender across different populations

**Cohorts**	**subtype of sCoVs**				
	**229E**	**HKU1**	**OC43**	**NL63**	**SARS-COV-2**
PHD	0.221	0.352	0.479	0.614	0.683
VD	0.067	0.413	0.947	0.092	0.947
